# Strategies for Metabolic Engineering of *Escherichia coli* for β-Carotene Biosynthesis

**DOI:** 10.3390/molecules31040611

**Published:** 2026-02-10

**Authors:** Jiali Liu, Yilin Shi, Daxin Zhao, Minghao Lin, Ping Wang, Ying Zhou, Xiaohui Yan

**Affiliations:** 1 National Key Laboratory of Chinese Medicine Modernization, State Key Laboratory of Component-Based Chinese Medicine, Tianjin University of Traditional Chinese Medicine, 10 Poyanghu Road, Tianjin 301617, China; liujiali2025@gmail.com (J.L.); shiyilin011024@163.com (Y.S.); zhaodaxin2001@gmail.com (D.Z.); 2 Haihe Laboratory of Modern Chinese Medicine, 10 Poyanghu Road, Tianjin 301617, China; 3 School of Chinese Materia Medica, Tianjin University of Traditional Chinese Medicine, 10 Poyanghu Road, Tianjin 301617, China; linminghao2002@gmail.com (M.L.); wangping81@tjutcm.edu.cn (P.W.)

**Keywords:** β-carotene, *E. coli*, metabolic engineering, MEP pathway, MVA pathway

## Abstract

β-Carotene has extensive applications in the food, pharmaceutical, and cosmetics industries. Traditional chemical synthesis methods face challenges such as byproduct residues and high costs, whereas natural extraction is constrained by low yields and complex processes. Recent advancements in synthetic biology and metabolic engineering have paved the way for the heterologous biosynthesis of β-carotene in microorganisms. Owing to its rapid growth, convenience of genetic manipulation, and suitability for producing apocarotenoids, *Escherichia coli* is an ideal host for the production of β-carotene and its derivatives, as exemplified by the record production of multiple apocarotenoids in engineered *E. coli* strains. Here, we summarize the metabolic engineering strategies employed to produce β-carotene in *E. coli*, including manipulation of the endogenous MEP pathway, introduction of the hybrid MVA pathway, modulation of central carbon metabolism, modification of the cell membrane, and fermentation process optimization. As β-carotene acts as a biosynthetic hub for many carotenoids and apocarotenoids, we also highlighted the importance of efficient β-carotene production for the sustainable preparation of these compounds. This review aims to provide theoretical insights for designing talented β-carotene producers and laying the foundation for the sustainable manufacturing of valuable carotenoids and apocarotenoids.

## 1. Introduction

Carotenoids are a class of C40 tetraterpenoids produced by plants, fungi, algae, and cyanobacteria [1]. They are responsible for the characteristic yellowish, reddish, and orange colors of fruits, vegetables, fish, birds, reptiles, and butterflies. Approximately 600 carotenoids are characterized in nature, of which approximately 40 are routinely consumed in human diets. Carotenoids typically feature an extended conjugated double-bond system flanked by cyclic end groups. Carotenoids are typically categorized into two main groups: hydrocarbon carotenoids (compounds without oxygen in their chemical formula), including lycopene, β-carotene, α-carotene, and xanthophylls (oxygen-containing carotenoids), such as lutein, β-cryptoxanthin, zeaxanthin, antheraxanthin, capsanthin, violaxanthin, and neoxanthin.

β-carotene is present in abundance in the human diet, found in foods such as apricots, cantaloupe, carrots, pumpkin, and sweet potatoes. It acts as a precursor to vitamin A, an essential nutrient involved in growth, vision, and cellular functions. β-Carotene exhibits numerous properties in the treatment of cancer, eye diseases, heart disorders, Alzheimer’s disease, neurodegenerative diseases, inflammation, osteoporosis [2,3]. Humans cannot synthesize β-carotene; therefore, they must consume β-carotene-containing vegetables and fruits to maintain normal physiological processes in the body. In addition to its pharmacological activities, β-carotene is used as a coloring agent in the food, beverage, pharmaceutical, and cosmetic industries [4,5]. Owing to the high demand for nutritional supplements and health products, the β-carotene market is rapidly expanding. According to a report from Global Market Insights Inc., the global β-carotene market is projected to be worth 610.5 million USD in 2024 and is estimated to reach 952.5 million USD in 2034, with a compound annual growth rate (CAGR) of 4.7% during this period. In nature, the degradation products of β-carotene, such as abscisic acid, β-ionone, and strigolactones (SLs), are important plant hormones involved in the regulation of plant physiology [6,7].

β-carotene on the market is majorly obtained by chemical synthesis. However, this method is now challenged by growing concerns regarding the use of chemically synthesized β-carotene as a food additive or cosmetic agent. This issue has reignited the enthusiasm for producing β-carotene via large-scale fermentation, which is regarded as a cost-effective and sustainable source for β-carotene production because the scaling-up process is less cumbersome than plant extraction and chemical synthesis. Natural carotenoid producers, such as algae (*Haematococcus pluvialis* [8], *Chlorella vulgaris* [9], and *Dunaliella algae* [10]), fungi (*Blakeslea trispora* [11], *Rhodotorula mucilaginosa* [12], *Phaffia rhodozyma* [13,14,15], and *Kluyveromyces marxianus* [16]), and bacteria (*Rhodococcus opacus*, *Rhodococcus erythropolis* [17,18]), are potential hosts for β-carotene fermentation (Table 1).

With advances in synthetic biology, the design and construction of β-carotene biosynthetic pathways in suitable chassis organisms, such as *Escherichia coli, Saccharomyces cerevisiae*, *Komagataella phaffii* (formerly *Pichia pastoris*), *Yarrowia lipolytica*, and *Vibrio natrigenes*, offer a green and sustainable solution for the large-scale production of β-carotene and its derivatives (Table 1) [19,20,21,22]. *Y. lipolytica* exhibits remarkable advantages in the accumulation of fat-soluble β-carotene due to its excellent lipid storage capacity. Currently, the maximum yield of β-carotene in engineered *Y. lipolytica* strains has reached 39.5 g/L, demonstrating great potential for further exploitation [23,24,25,26]. *K. phaffii* can achieve biomass of >100 g/L (dry cell weight) in optimized fed-batch fermentation, which is significantly higher than that of *E. coli*. Recent metabolic engineering of *K. phaffii* has produced 10.2 g/L of lycopene [27,28].

Although current efforts for β-carotene production prefer yeast hosts, *E. coli* remains a superior chassis for the efficient production of β-carotene and its derivatives, including ketocarotenoids, apocarotenoids, and oxygenated carotenoids. On one hand, *E. coli* can produce up to 3.93 g/L of β-carotene by continuous fermentation, which is comparable to the β-carotene titers in yeasts [29]. *E. coli* has also been employed to produce other carotenoids. More importantly, *E. coli* can efficiently express carotene-modifying enzymes, exemplified by the [2Fe-2S]-containing β-carotene isomerase DWARF27 (D27) and carotenoid cleavage dioxygenases (CCDs). It is well recognized that the functional expression of these enzymes in yeast is challenging. Compared to yeast, *E. coli* exhibits greater tolerance to oxygenated carotenoid intermediates and apocarotenoids, which often impose oxidative or membrane stress in eukaryotic hosts. The advantages of using *E. coli* as a chassis for the microbial fermentation of valuable apocarotenoids have been previously demonstrated [19]. To achieve high-yield β-carotene production in *E. coli*, researchers have employed diverse strategies, including metabolic, enzyme, and membrane engineering, and fermentation optimization. This paper systematically reviews heterologous β-carotene biosynthesis methods in *E. coli*, focusing on metabolic engineering strategies for yield improvement, and aims to provide theoretical and technical references for the efficient production of β-carotene and carotenoid derivatives in recombinant *E. coli* strains.

**Table 1:** Natural hosts and common chassis for carotenoid production.

Producer Type	Strain	Compounds	Ref.
Natural producers	*Haematococcus pluvialis*	Astaxanthin	[8]
	*Chlorella vulgaris*	Astaxanthin	[9]
	*Dunaliella salina*	β-carotene	[10]
	*Blakeslea trispora*	lycopene, β-carotene	[11]
	*Rhodotorula mucilaginosa*	β-carotene, astaxanthin	[12]
	*Phaffia rhodozyma*	β-carotene, astaxanthin	[13,14,15]
	*Kluyveromyces marxianus*	Astaxanthin	[16]
	*Rhodococcus* sp.	lycopene, γ-carotene	[17,18]
Heterologous producers	*Saccharomyces cerevisiae*	lycopene, β-carotene, astaxanthin	[20,21,22]
	*Yarrowia lipolytica*	lycopene, β-carotene, astaxanthin	[23,24,25,26]
	*Komagataella phaffii*	lycopene, β-carotene	[27,28]
	*Escherichia coli*	lycopene, β-carotene, astaxanthin	[29,30,31,32]

## 2. Metabolic Engineering Strategies for β-Carotene Production in *E. coli*

### 2.1. The β-Carotene Biosynthetic Pathway

**Figure 1:**
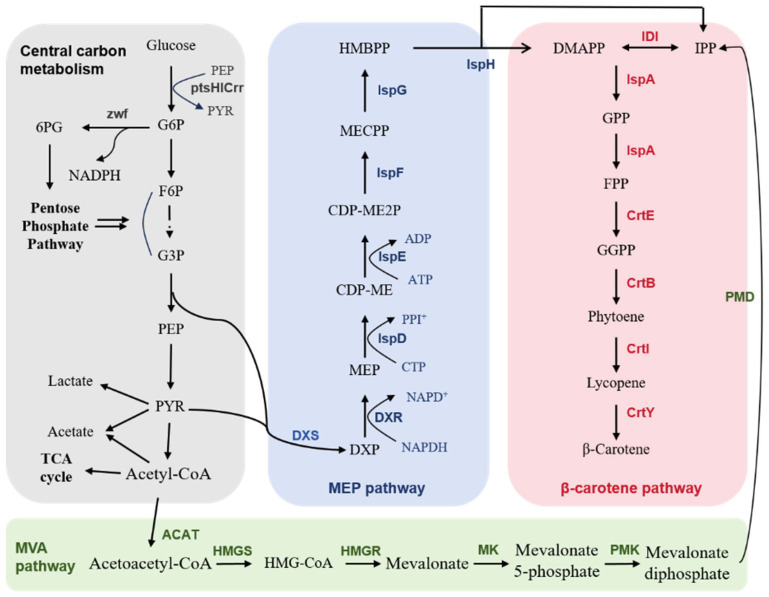
Biosynthetic pathways of β-carotene. Abbreviation: F6P, fructose-6-phosphate; G3P, glyceraldehyde-3-phosphate; G6P, glucose-6-phosphate; PEP, phosphoenolpyruvate; PYR, pyruvate; HMG-CoA, 3-hydroxy-3-methyl-glutaryl-CoA; DXP, 1-deoxy-D-xylulose-5-phosphate; MEP, 2-methyl-D-erythritol-4-phosphate; CDP-ME, 4-diphosphocytidyl-2-*C*-methyl-D-erythritol; CDP-ME2P, 4-diphosphocytidyl-2-*C*-methyl-D-erythritol-2-phosphate; MECPP, 2-*C*-methyl-D-erythritol 2,4-cyclodiphosphate; HMBPP, 1-hydroxy-2-methyl-2-E-butenyl-4-diphosphate; DMAPP, dimethylallyl pyrophosphate; IPP, isopentenyl pyrophosphate; GPP, geranyl pyrophosphate; FPP, farnesyl pyrophosphate; GGPP, geranylgeranyl pyrophosphate; CTP, cytidine triphosphate; PPI^+^, pyrophosphate; ATP, adenosine triphosphate; ADP, adenosine diphosphate; NAD^+^, nicotinamide adenine dinucleotide; NADP^+^, nicotinamide adenine dinucleotide phosphate; NADPH, nicotinamide adenine dinucleotide phosphate reduced; Zwf, G6P dehydrogenase; Dxs, DXP synthase; Dxr, DXP reductoisomerase; IspD, MEP cytidylyltransferase; IspE, CDP-ME kinase; IspF, MECPP synthase; IspG, HMBPP synthase; ACAT, acetoacetyl-CoA acyltransferase; HMGS, HMG-CoA synthase; HMGR, HMG-CoA reductase; MK, mevalonate kinase; PMK, phosphomevalonate kinase; PMD, mevalonate-PP decarboxylase; IDI, Isopentenyl-diphosphate isomerase; ISPA, FPP synthase; CrtE, GGPP synthase; CrtB, phytoene synthase; CrtI, phytoene dehydrogenase; CrtY, lycopene β-cyclase.

The biosynthetic pathway of β-carotene and its associated genes have been extensively studied in various species [33]. Similar to other terpenoids, β-carotene is biosynthesized from isopentenyl pyrophosphate (IPP) and dimethylallyl pyrophosphate (DMAPP). In eukaryotic cells, including plant cytoplasm and mitochondria, these two precursors are synthesized via the mevalonate (MVA) pathway. In bacteria and plant plastids, IPP/DMAPP are biosynthesized via the methylerythritol phosphate (MEP) pathway (Figure 1). The MEP pathway begins with the formation of 1-deoxy-D-xylulose-5-phosphate (DXP), catalyzed by DXP synthase (DXS) from pyruvate (PYR) and glyceraldehyde-3-phosphate (G3P). DXP is converted to IPP and DMAPP in six sequential steps. IPP and DMAPP are interconverted by IPP isomerase (IDI). DXS and IDI are speed-limiting enzymes in the MEP pathway. The MVA pathway begins with the formation of acetoacetyl-CoA from two acetyl-CoA molecules by acetoacetyl-CoA acyltransferase (ACAT). Acetoacetyl-CoA is then converted by 3-hydroxy-3-methylglutaryl-CoA synthetase (HMGS) to generate 3-hydroxy-3-methylglutaryl-CoA (HMG-CoA), and then by HMG-CoA reductase (HMGR) to afford MVA. MVA is converted to IPP in three sequential steps catalyzed by MVA kinase (MK), phosphomevalonate kinase (PMK), and MVA pyrophosphate decarboxylase (PMD). The condensation of IPP and DMAPP leads to the formation of geranyl pyrophosphate (GPP). GPP is then condensed with IPP to form farnesyl pyrophosphate (FPP) via FPP synthase (IspA). Geranylgeranyl pyrophosphate (GGPP) is formed by the condensation of one molecule each of FPP and one molecule of IPP by GGPP synthase (CrtE). Two molecules of GGPP are coupled by phytoene synthase (CrtB) to form phytoene, which is converted to lycopene by phytoene desaturase (CrtI). Lycopene has 11 conjugated double bonds and 2 non-conjugated double bonds. Finally, lycopene is cyclized by lycopene cyclase (CrtY) to form β-carotene [34].

The overall cofactor consumption and mass yield for synthesizing IPP from glucose via the MVA (Equation (1)) and MEP pathways (Equation (2)) have been compared in detail [35]. The synthesis of one molecule of IPP/DMAPP via the MVA pathway consumes 1.5 molecules of glucose and four molecules of NAD(P)H. In contrast, the MEP pathway requires one molecule of glucose, two molecules of NADPH, and three molecules of ATP to generate one molecule of IPP or DMAPP. When the synthesis of NAD(P)H and ATP via the central carbon pathway is included, the MEP pathway is predicted to have a theoretical mass yield of 30.2% from glucose, whereas that of the MVA pathway is 25.2%. In summary, the MVA pathway has a lower cofactor requirement, whereas the MEP pathway has a higher theoretical mass yield.

(1) $$1.5\;Glucose+2NADPH+6NAD=IPP+{4CO}_{2}+6NADH+2NADP$$

(2) $$Glucose+3NADPH+NAD+3ATP=IPP+{CO}_{2}+3ADP+NADH+3NADP$$

### 2.2. Introduction of β-Carotene Biosynthetic Genes

**Figure 2:**
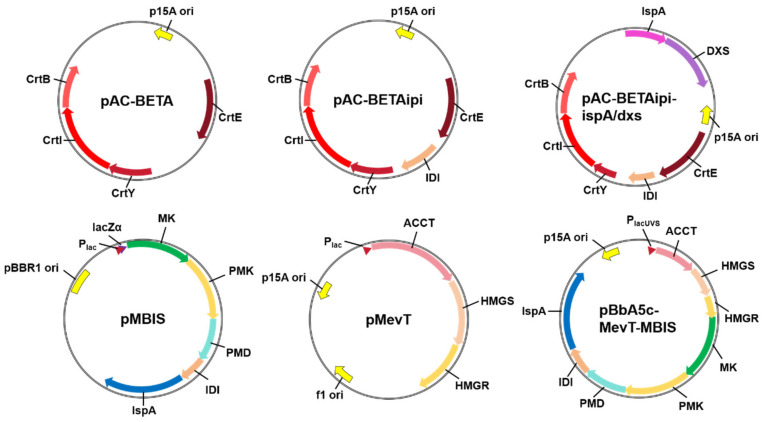
Plasmids harboring genes of the β-carotene biosynthetic (**top**) and MVA (**bottom**) pathways.

The *crt* gene clusters responsible for β-carotene biosynthesis have been extensively studied in bacteria of the Actinomycetota, Bacillota, Bacteroidota, Chlorobiota, and Pseudomonadota phyla [33]. *E. coli* does not naturally possess β-carotene biosynthetic genes; therefore, introducing a *crt* gene cluster into the host is the primary step in achieving de novo synthesis of β-carotene. *crt* gene clusters from *Erwinia uredovora*, *Erwinia herbicola*, *Agrobacterium aurantiacum*, *Pantoea ananatis*, and Deinococcus radiodurans have been cloned and validated in *E. coli* [36,37,38,39]. The plasmid pAC-BETA, harboring *crtE*, *crtB*, and *crtI* from *E*. *herbicola* and *crtY* from *Arabidopsis*, was constructed and used for the de novo biosynthesis of β-carotene in *E. coli* (Figure 2) [36,40,41]. Insertion of the *E. coli*-derived *ipi* and *ispA*/*dxs* genes into pAC-BETA resulted in the formation of plasmids pAC-BETA*ipi* and pAC-BETA*ipi*-*ispA*/*dxs*. These two plasmids enhanced the expression of rate-limiting enzymes and balanced the IPP/DMAPP ratio, thus significantly increasing the β-carotene titer in *E. coli* [42,43]. Plasmid pT-DHB, which contains *crtE*, *crtB*, and *crtI* from *Pantoea agglomerans* and *crtY* from *Pantoea ananatis*, was constructed for synthesizing carotenoids in *E. coli* [44,45]. Plasmids pTB-EIBY (high-copy) and pTB-EIBYrop (low-copy), containing the *crt* gene cluster from *E. uredovora*, were constructed for the overproduction of β-carotene in *E. coli* [46]. In addition to plasmid-borne carotenogenic genes, the *crtEXYIB* gene operon from *P. agglomerans* was integrated into the *E. coli* chromosome. After combined engineering of the MEP and central metabolic pathways, the optimized *E. coli* strain produced 2.1 g/L β-carotene [31].

In addition to *crt* genes from bacteria, eukaryotic carotenogenic genes have been introduced into *E. coli* for β-carotene production. The expression of seven genes (*DsGGPS*, *DsPSY*, *DsPDS*, *DsZISO*, *DsZDS*, *DsCRTISO*, and *DsLYCB*) from the microalgae *Dunaliella salina* in *E. coli* enabled the production of a series of carotenoids [47]. CrtYB from *Xanthophyllomyces dendrorhous* catalyzes the synthesis of phytoene from GGPP and the cyclization of lycopene into β-carotene. Co-expression of the *XdcrtYB* gene operon with other carotenogenic genes in *E. coli* led to β-carotene production of [48].

During β-carotene production in engineered *E. coli*, a clear trade-off exists between biomass formation and product synthesis, as both processes compete for the same intracellular resource. Multiple key precursors, such as G3P, PYR, and acetyl-CoA, which are required for amino acid, nucleotide, and lipid biosynthesis, are redirected towards carotenoid production. Consequently, the supply of building blocks for cell growth is limited. β-Carotene synthesis also imposes a high energetic and redox cost, consuming substantial amounts of ATP and NADPH, which further constrains growth-associated metabolism. The overexpression of heterologous enzymes reallocates ribosomal and transcriptional capacity and creates an additional proteomic burden on the host. Moreover, the accumulation of hydrophobic carotenoids in the membrane perturbs the membrane structure and respiration, induces oxidative stress, and reduces cellular fitness. These combined effects typically lead to slower growth rates, lower biomass formation, and diminished culture robustness as production increases. Therefore, high titers are rarely achieved simultaneously with rapid cell growth. Effective industrial strategies aim to decouple these phases by first maximizing biomass and then activating carotenoid synthesis through dynamic regulation, balanced precursor and cofactor supply, and tolerance or membrane engineering to alleviate toxicity.

### 2.3. Manipulation of the Endogenous MEP Pathway

An efficient supply of IPP and DMAPP precursors is crucial for β-carotene production in *E. coli*. Two strategies are often adopted to increase the metabolic flux via the MEP pathway in *E. coli*: (1) increasing the production of endogenous enzymes and (2) introducing exogenous enzymes with higher catalytic activity. The concomitant overexpression of *dxs*/*idi* or *dxr*/*idi* in *E. coli* increased carotenoid production by 3.5-fold [49]. Replacement of the native promoters of the chromosomal genes of the MEP pathway with the strong T5 promoter increased β-carotene production 24.5-fold, with a titer of 6 mg/g dry cell weight (CDW) [50]. Although *dxs* and *dxr* are the two rate-limiting enzymes of the MEP pathway in *E. coli*, their overexpression with a strong inducible promoter or high-copy plasmid could lead to decreased cell growth and lower β-carotene production [51]. In the second strategy, critical genes of the MEP pathway from other bacteria are often introduced into *E. coli*. By introducing *dxs* genes from *Bacillus subtilis* or *Synechocystis* sp. 6803 into *E. coli*, the lycopene titer in the resultant strains was doubled compared to that of the control strain [52]. The incorporation of an engineered *E. coli* strain harboring *B. subtilis*-derived DXS and IPI and *Abies grandis*-derived GPPS2 achieved a β-carotene yield of 15.2 mg/L, representing a 14-fold enhancement compared to the control strain [37]. Heterologous expression of *dxs* and *ispA* from *Vibrio* sp. Dhg in *E. coli* resulted in a 1.88-fold increase in lycopene production [53].

### 2.4. Introduction of Exogenous MVA Pathway

In addition to manipulating the endogenous MEP pathway, the introduction of the MVA pathway from eukaryotic organisms into *E. coli* represents a more efficient method for increasing carotenoid production [54]. The *S. cerevisiae* MVA pathway was reconstituted in two plasmids: pMevT (harboring genes encoding AACT, HMGS, and tHMGR) and pMBIS (harboring genes encoding MK, PMK, PMD, IDI, and IspA) (Table 2). *E. coli* strains containing these two plasmids demonstrated broad applicability as a universal host for terpenoid biosynthesis upon the introduction of terpene synthase genes [54]. Subsequently, the plasmid pBbA5c-MevT-MBIS, which harbors genes from pMevT and pMBIS, was constructed to enhance the MVA pathway. For example, an engineered *E. coli* strain harboring similar plasmids produced β-farnesene at a titer of 8.74 g/L [54]. The *mvaK1*, *mvaK2*, *mvaD*, and *idi* genes from *Streptococcus pneumoniae* were cloned into a lycopene-producing *E. coli* strain (pT-LYCm4) to obtain *E. coli* (pT-LYCm4/pSSN12Didi). Under optimized conditions with MVA supplementation, the resulting strain produced 102 mg/L (22 mg/g DCW) of lycopene [55]. A more efficient MVA pathway was constructed in *E. coli* through the modular assembly of the *Enterococcus faecalis* upper pathway and the *S. pneumoniae* lower pathway. This integrated system enabled robust β-carotene production (465 mg/L) in the optimized strain without MVA supplementation [42]. Similarly, the top MVA pathway module from *E. faecalis* and the bottom module from *S. cerevisiae* were reconstituted in *E. coli*. By further introducing the HMGS^A110G^ mutant, an isoprene yield of 6.3 g/L was achieved via fed-batch fermentation [56]. Integration of this hybrid MVA pathway into an MEP-optimized *E. coli* strain resulted in β-carotene production of 122.4 ± 6.2 mg/L in shake flasks [37]. The incorporation of genes encoding MK, PMK, and PMD from *Enterococcus faecium* VTCC-B-935 into *E. coli* enhanced β-carotene production by 3-fold (17.7 mg/L) [57].

To ensure stable expression of exogenous MVA genes and circumvent the metabolic burden imposed by the plasmid, MVA pathway genes are often integrated into the chromosome of *E. coli*. By Integrating the MVA pathway from *S. cerevisiae* into the *E. coli* chromosome and modulating the MVA pathway with various promoters and ribosomal binding sites (RBSs), β-carotene production was increased by 51% [58]. It has been demonstrated that integrating MVA modules at multiple chromosomal loci of *E. coli* is more efficient than using a plasmid carrying the MVA pathway for lycopene production [59].

### 2.5. Modification of the Central Metabolic Module

G3P and PYR are key intermediates of glycolysis and precursors of the MEP pathway. Efficient synthesis of β-carotene also requires a sufficient supply of NADPH and ATP cofactors. Therefore, the central metabolism of *E. coli* is often modified to increase the supply of key intermediates and cofactors to enhance β-carotene production (Figure 1). Deletion of genes encoding enzymes utilizing these intermediates or cofactors, such as ZWF, GdhA, YjgB, and Pgi, can increase the precursor or cofactor supply, thus ensuring more β-carotene formation [60,61,62,63]. In contrast, overexpression of enzymes enhancing precursor and cofactor supply, such as NadK, POS5, PntAB, SthA, Mdh, GalP, Glk, has been conducted to boost β-carotene production [61]. Overexpression of genes in the central metabolic modules, including *sucAB*, *sdhABCD*, and *talB*, increased the lycopene yield by 76% and achieved a lycopene titer of 3.52 g/L in fed-batch fermentation [64]. The combined deletions of *gdhA*, *zwf*, *yjgB*, and *ptsHIcrr* and overexpression of *nadK* in *E. coli* ZF43 strain enabled the production of 266.4 mg/L β-carotene in shake flasks and 2.6 g/L in a 5-L bioreactor [61].

### 2.6. Balancing the Gene Expression Levels

An efficient cell factory requires a delicate balance between cellular growth and product accumulation. It is expected that metabolic flux, energy, and cofactors will be optimally distributed to sustain biomass formation and maximize target compound biosynthesis. Excessive resources for cell growth can limit product yield, whereas premature or excessive product formation can impose a metabolic burden, inhibit cell viability, and eventually reduce product yield. Therefore, the dynamic regulation of metabolic pathways, coordinated control of gene expression, and rational allocation of carbon and cofactor fluxes are essential for achieving a robust trade-off that enhances overall productivity, titer, and process stability in industrial biomanufacturing systems. Multiple biosynthetic intermediates of the MEP pathway (HMBPP, DXP, MEP, CDP-ME, and MECPP) [61,65,66], MVA pathway (HMG-CoA, MVA, and Mevalonate 5-phosphate) [61,67], and downstream pathway (IPP, DMAPP, and FPP) [66] are toxic to *E. coli* cells or exert inhibitory effects on enzymes. As demonstrated by the balanced expression of *ispG* and *ispH* [68] and *idi* and *ispA* [69], the balanced expression of these enzymes prevented the accumulation of toxic intermediates, allowed sufficient *E. coli* growth, and maximized the flux towards β-carotene. Several novel techniques have been adopted to balance gene expression in *E. coli*, such as assembling the last enzyme of the MVA pathway with the first enzyme of the carotenoid pathway with RIAD and RIDD short peptide tags [70], employing RBS library screening to balance the expression levels of *crtW* and *crtZ* [71], designing diverse 5′-UTR sequences [72], and constructing a plasmid library with various regulatory elements to modulate the expression of MVA pathway genes (*AACT*, *HMGS*, *MK*, *PMK*, and *PMD*) [73].

### 2.7. Engineering the Membrane Architecture

As a hydrophobic compound, β-carotene tends to accumulate within the cell membranes, reducing membrane fluidity, inducing cytotoxicity, and limiting productivity. Various strategies, such as adjusting membrane lipid composition and permeability, engineering membrane proteins, optimizing membrane morphology, and modifying inner-membrane vesicles (IMVs) and outer-membrane vesicles (OMVs), have been applied to increase the production of hydrophobic metabolites [74]. Overexpression of AlMGS, the monoglycosyldiacylglycerol synthase from *Acholeplasma laidlawii* in *E. coli*, significantly increased the intracellular membrane vehicles and enhanced β-carotene production by 39% [75]. The co-expression of AlMGS with enzymes responsible for diglyceride-3-phosphate synthesis (PlsB and PlsC) in *E. coli* promoted cell membrane synthesis and increased lycopene production by 32% [76]. Wu et al. established an artificial membrane vesicle transport system (AMVTS) in *E. coli* by knocking out *tolR* and *nlpI* and overexpressing *AccABCD* and *PlsBC* genes. AMVTS significantly facilitated β-carotene secretion and led to a 3.2-fold increase in productivity. The introduction of AVMTS into the β-carotene hyperproducing strain CAR025 increased the production from 27.7 to 44.8 mg/g in shake flasks [77]. Membrane expansion strategies have also been adopted for the efficient production of β-carotene [72]. Co-expression of *cav1* (encoding human caveolin-1) and *plsBC* promoted IMVs formation, whereas knocking down rffD and rfaD enhanced OMV secretion. The concerted expansion of the cell membrane enabled the production of 343 mg/L of β-carotene in *E. coli* [72].

### 2.8. Other Factors Affecting β-Carotene Production

Fermentation conditions, including medium composition, pH, temperature, and cultivation mode, critically influence β-carotene production. Numerous studies have shown that glycerol is a superior carbon source for β-carotene production [54,55,57,62,78,79]. For instance, Guo et al. introduced aldehyde reductase (*alrD*) and aldehyde dehydrogenase (*aldH*) genes from *Ralstonia eutropha* H16 into *E. coli* to establish a glycerol-utilization pathway and improved β-carotene titer by 50% [79]. Liu et al. introduced a fatty acid transport system to facilitate the use of hydrophobic substrates for lycopene synthesis. The introduction of fatty acid transport and metabolic systems into *E. coli* facilitated lycopene production from waste cooking oil. The engineered strain produced 2.7 g/L lycopene using glucose and hydrolyzed waste cooking oil as carbon sources [9]. It was also showed that β-carotene synthesis occurred exclusively at neutral pH, whereas alkaline conditions inhibited the conversion of lycopene to β-carotene [43].

Compared to fermentation in shake flasks, high-density fermentation in bioreactors enables real-time monitoring and control of critical fermentation parameters, such as pH, temperature, dissolved oxygen (DO), and substrate concentrations, thus ensuring optimal cellular growth, minimizing metabolic stress, and improving carbon flux toward β-carotene biosynthesis. As a result, the β-carotene titer can be significantly increased by fed-batch fermentation in fermenters [31,61,72]. The host strain also has a substantial effect on β-carotene production [77]. Different *E. coli* strains (DH5α, XL1-Blue, and JM101) exhibited significantly different lycopene production, with the XL1-Blue strain demonstrating the highest titer [51]. In another study, β-carotene production in various *E. coli* strains (MG1655, DH5α, S17-1, XL1-Blue, and BL21) was compared, and DH5α was identified as the optimal producer [45].

**Figure 3:**
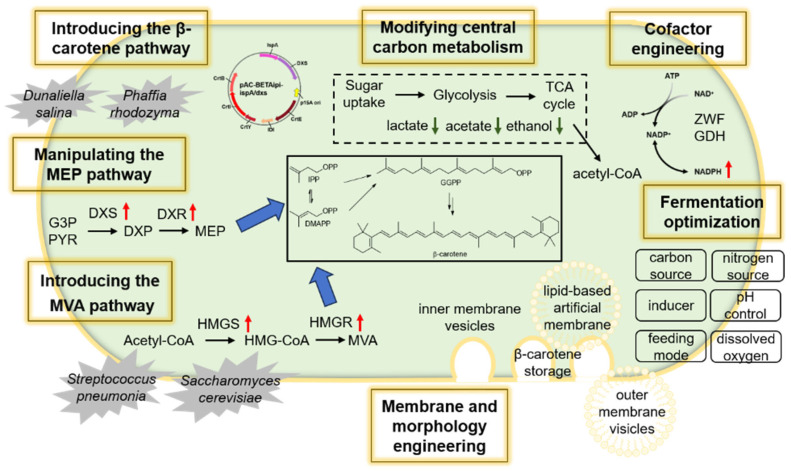
Strategies used to enhance β-carotene production in *E. coli*.

In Figure 3 and Table 2, we summarize representative studies reporting high β-carotene titers in engineered *E. coli* strains, highlighting the key metabolic engineering strategies and specific fermentation conditions that enabled efficient production. To the best of our knowledge, the current record titer for β-carotene production in *E. coli* is 3.93 g/L, as recently achieved by Ji et al. [29] They combined multiple strategies, including membrane engineering (overexpression of fatty acid biosynthetic genes, deletion of regulatory genes involved in outer membrane channels and cell division), stepwise engineering of the MVA pathway and central metabolism, and fine-tuning of gene expression levels. The fermentation operation was divided into a three-stage semi-continuous process. Using this transitional-state fermentation method, the final β-carotene titer achieved was 3.93 g/L, which was 23.2% higher than that with single-stage fermentation. The key message of this study is that cellular robustness is essential for efficient β-carotene production in *E. coli*.

**Table 2:** Representative β-carotene titers in engineered *E. coli* strains.

Chassis	Bioreactor	Titer (g/L)	Primary Metabolic Engineering Strategies	Specific Fermentation Conditions	Ref.
MG1655	3-L fermenter	3.93	MVA pathway, membrane engineering, dynamic regulation of *mvaE* expression	Three-stage semi-continuous fermentation	[29]
BL21(DE3)	5-L fermenter	3.20	Optimized MEP pathway with exogenous *dxs* and *gpps2* genes, hybrid MVA pathway.	Glycerol feeding	[41]
MG1655	5-L fermenter	2.58	Deletion of *gdhA*, *zwf*, *phtHIcrr*, and *yjgB*, overexpression of *nadK* on a low-copy number plasmid.	Fed-batch fermentation, modified minimal medium with trace metal solution	[61]
DH5α	7-L fermenter	2.47	Co-expression of pT-DHB (contains downstream genes for β-carotene biosynthesis) and pS-NA (contains the exogenous MVA pathway)	Chemically defined medium with amino acid supplementation, glycerol feeding	[80]
ATCC 8739	7-L fermenter	2.1	Combined engineering of the MEP, β-carotene biosynthesis, and central metabolic modules.	Feeding of glycerol, peptone, yeast extract, and MgSO4·7H_2_O	[31]
BW25113	5-L fermenter	1.9	Optimizing CrtY expression using an oligo-linker mediated DNA assembly (OLMA) library	Fed-batch fermentation	[81]
DH5α	3.4-L fermenter	0.66	Co-expression of pT-DHB and pS-NA	Feeding glycerol, yeast extract, MgSO4·7H_2_O, and thiamine	[82]
DH5α	shake flask	0.50	Co-expression of pT-DHB and pSSN12Didi (contains downstream genes of the MVA pathway)	Fermentation at 29 °C in 2YT medium for 144 h, addition of 16.5 mM mevalonate and glycerol	[44]

## 3. Significance of β-Carotene Production in *E. coli*

**Figure 4:**
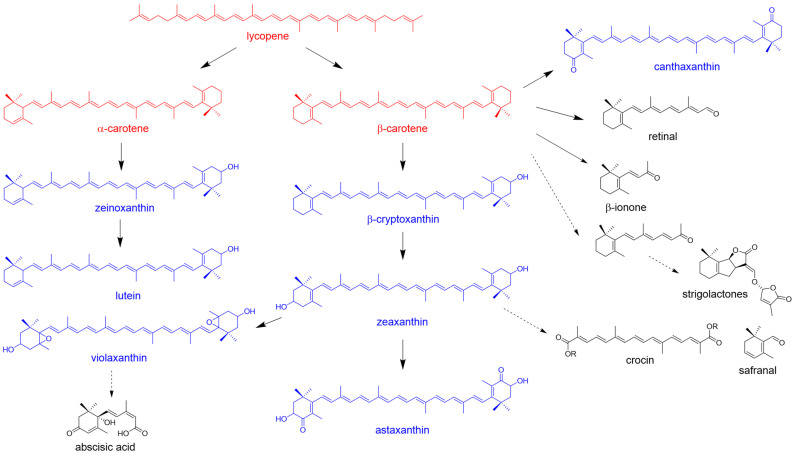
Metabolic pathways of carotenoids (red), xanthophylls (blue), and apocarotenoids (black).

Studies on β-carotene production in *E. coli* have facilitated the fermentation of other carotenoids and apocarotenoids (Figure 4). Apocarotenoids constitute a diverse family of hormones, pigments, nutraceuticals, fragrances, and pharmaceuticals with exceptionally high value. Many of these compounds are produced in trace amounts in plants (e.g., saffron, rose, and tobacco), making their extraction inefficient and environmentally burdensome. Therefore, the reconstitution of the biosynthetic pathways of apocarotenoids in an efficient β-carotene-producing *E. coli* strain is a promising method for the sustainable production of these valuable compounds.

In nature, β-carotene mainly exists in the all-*trans*- and 9-*cis*-forms. The *all*-*trans* isomer is the most prevalent form, whereas 9-*cis*-β-carotene is predominantly found in certain plants and algae. *all*-*trans*-Carotene is the precursor of several valuable natural products, such as Vitamin A and β-ionone. 9-*cis*-β-Carotene is the precursor for all SLs [83]. The biosynthetic pathways of most SLs have been elucidated [84]. *All-trans*-β-carotene is isomerized by β-carotene isomerase D27 to generate 9-*cis*-β-carotene, which is subsequently cleaved by CCD7 and CCD8 to yield carlactone (CL). CL is further oxidized by CYP450 oxygenases to produce various SLs. Recently, the biosynthesis of SLs, including CL, carlactonoic acid (CLA), 5-deoxystrigol (5DS), and 4-deoxyorobanchol (4DO), was accomplished using *E. coli* and *S. cerevisiae* consortia [43]. This study lays the foundation for sustainable SL production.

Astaxanthin exhibits exceptional antioxidant capacity and is one of the most potent, naturally occurring antioxidants. It has broad applications in the pharmaceutical, cosmetic, food additive, and aquaculture industries. Astaxanthin can be converted from β-carotene by β-carotene hydroxylase (CrtZ) and ketolase (CrtW) [85,86]. The co-expression of CrtW from *Brevundimonas* sp. SD212 and CrtZ from *Pantoea ananatis*, or both enzymes from *Adonis aestivalis* in β-carotene-producing *E. coli* strains can produce astaxanthin [87,88]. Zeaxanthin plays a critical role in human nutrition and metabolism due to its antioxidant properties. Efficient zeaxanthin production (19.5 mg/g DCW) in *E. coli* was achieved using a combined approach, including gene fusion, coordinated gene expression, promoter optimization, chromosomal integration, and comparative analysis of key enzyme genes from different species [89]. In plants, the conversion of zeaxanthin into abscisic acid (ABA) is catalyzed by 9-*cis*-epoxy-carotenoid dioxygenase (NCED); however, ABA production in *E. coli* has not yet been achieved [90].

Retinoids are essential for vision and dermatological therapies. A retinal titer of 345.7 mg/L was achieved in an engineered *E. coli* BL21(DE3) strain using a combination of directed evolution, RBS optimization, and membrane engineering [91]. Crocins and crocetin are valuable natural colorants and antioxidants with neuroprotective properties. The microbial production of crocetin in *E. coli* (34.77 mg/L) was achieved by batch fermentation in a 5-L bioreactor [92]. Volatile compounds, such as β-ionone, damascenone, and safranal, dominate the global flavor and fragrance market because of their ultra-low odor thresholds and premium sensory qualities. A recent review systematically summarized the production of these volatile compounds in microbial cell factories [93]. Notably, a record titer of α-ionone (~700 mg/L) was achieved in an engineered *E. coli* strain by optimizing the metabolic pathway and reducing oxidative stress from H_2_O_2_ [94]. These successful examples strongly support the feasibility of using engineered *E. coli* for the sustainable and economical production of valuable apocarotenoids.

## 4. Conclusions and Prospect

Compared to yeast hosts, engineered *E. coli* offers several advantages for carotenoid and apocarotenoid production. Carbon flux can be routed directly to IPP/DMAPP through the cytosolic MEP or heterologous MVA pathways without organelle transport barriers or obligatory lipid storage, enabling higher precursor efficiency. Its metabolism has been quantitatively characterized and supported by highly predictive genome-scale models, allowing rational flux balancing and reducing empirical optimization. Moreover, many plant carotenoid-cleaving dioxygenases and glycosyltransferases function readily in bacterial cytosol, facilitating apocarotenoid pathway reconstruction with fewer folding and compartmentalization constraints than in yeast. Chromosomal integration of hybrid MVA modules, CRISPR-Cas9–assisted genome editing [95], and systematic fermentation optimization have improved strain stability and productivity [96,97]. In addition, protein engineering and spatial organization of pathway enzymes increase catalytic efficiency and reduce intermediate losses [98,99]. Together, these features make *E. coli* a versatile platform for scalable carotenoid biosynthesis. Efficient β-carotene fermentation in *E. coli* has laid the foundation for the production of valuable carotenoids and apocarotenoids, such as lutein, astaxanthin, zeaxanthin, canthaxanthin, ABA, and SLs [100].

Despite these advantages, a persistent trade-off between cell growth and carotenoid overproduction remains a central challenge. β-Carotene synthesis competes directly with biomass formation for carbon, ATP, and NADPH, thus reducing the availability of precursors required for cell growth. The high expression of heterologous enzymes also imposes a significant proteomic burden. Carotenoids are hydrophobic molecules that accumulate in cell membrane. High β-carotene concentration disrupts membrane integrity and respiration. These stresses reduce cellular fitness and limit the cell density. Consequently, strains that produce high titers often display impaired growth and poor fermentation robustness, which constrain industrial productivity.

Emerging technologies in metabolic engineering provide new opportunities to overcome the growth–production conflict. Dynamic regulatory systems based on genetically encoded biosensors and CRISPRi/CRISPRa modules can adjust pathway expression in response to intracellular states, allowing biomass accumulation before activating carotenoid synthesis [101,102,103]. Genome-scale metabolic models and flux balance analysis can identify optimal gene targets and predict flux redistribution strategies, thereby guiding rational pathway design [104,105]. Computational and machine-learning-assisted optimization further accelerates strain development by prioritizing beneficial enzyme combinations and expression levels [106]. Moreover, tolerance engineering through membrane remodeling and antioxidant defense can mitigate carotenoid-induced toxicity. Advanced bioprocess strategies, including in situ product removal and semi-continuous fermentation, can enhance cellular robustness and productivity. The combination of dynamic control, genome-scale metabolic modeling, robustness engineering, and process innovation will enable next-generation *E. coli* cell factories with higher titers, improved stability, and scalable production to meet commercial demands for carotenoids and apocarotenoids.

## Data Availability

No new data were created or analyzed in this study.
